# A prospective cohort study of biopsychosocial factors associated with childhood urinary incontinence

**DOI:** 10.1007/s00787-018-1193-1

**Published:** 2018-07-06

**Authors:** Carol Joinson, Mariusz T. Grzeda, Alexander von Gontard, Jon Heron

**Affiliations:** 1 Population Health Sciences, Bristol Medical School, University of Bristol, Oakfield House, Oakfield Grove, Clifton, Bristol, BS8 2BN UK; 2 Department of Child and Adolescent Psychiatry, Saarland University Hospital, Homburg, Germany

**Keywords:** Child urinary incontinence, Bedwetting, Daytime wetting, Developmental trajectory, Prospective cohort, ALSPAC

## Abstract

The objective of the study was to examine the association between biopsychosocial factors and developmental trajectories of childhood urinary incontinence (UI). We used developmental trajectories (latent classes) of childhood UI from 4–9 years including bedwetting alone, daytime wetting alone, delayed (daytime and nighttime) bladder control, and persistent (day and night) wetting (*n* = 8751, 4507 boys, 4244 girls). We examined whether biopsychosocial factors (developmental level, gestational age, birth weight, parental UI, temperament, behaviour/emotional problems, stressful events, maternal depression, age at initiation of toilet training, constipation) are associated with the trajectories using multinomial logistic regression (reference category = normative development of bladder control). Maternal history of bedwetting was associated with almost a fourfold increase in odds of persistent wetting [odds ratio and 95% confidence interval: 3.60 (1.75–7.40)]. In general, difficult temperament and behaviour/emotional problems were most strongly associated with combined (day and night) wetting, e.g. children with behavioural difficulties had increased odds of delayed (daytime and nighttime) bladder control [1.80 (1.59–2.03)]. Maternal postnatal depression was associated with persistent (day and night) wetting [2.09 (1.48–2.95)] and daytime wetting alone [2.38 (1.46–3.88)]. Developmental delay, stressful events, and later initiation of toilet training were not associated with bedwetting alone, but were associated with the other UI trajectories. Constipation was only associated with delayed bladder control. We find evidence that different trajectories of childhood UI are differentially associated with biopsychosocial factors. Increased understanding of factors associated with different trajectories of childhood UI could help clinicians to identify children at risk of persistent incontinence.

**Electronic supplementary material:**

The online version of this article (10.1007/s00787-018-1193-1) contains supplementary material, which is available to authorized users.

## Introduction

Two major groups of childhood urinary incontinence (UI) can be differentiated: nocturnal enuresis (bedwetting) and daytime urinary incontinence (daytime wetting), which can co-occur. Both groups are heterogeneous and comprise different conditions with specific signs and symptoms, as delineated by the International Children’s Continence Society [1]. The aetiology of UI is believed to be multifactorial, involving a complex interrelationship of biological, genetic and psychosocial factors. Factors associated with childhood UI include family history [2–5], developmental and obstetric factors [2–7], behaviour/emotional problems [8–10], stress [11, 12], maternal psychopathology [10], toilet training [13–16] and constipation [17–19]. Prospective cohort studies focus either on bedwetting [2, 20–22] or daytime wetting [23]. Bedwetting alone and combined (day and night) wetting are thought to have differing underlying pathophysiological mechanisms [24, 25], but the extent to which factors are differentially associated with different types of childhood UI is currently not well understood. Studies have found higher levels of developmental and obstetric factors [3–5] and psychological problems [26, 27] in children with combined wetting compared with bedwetting alone. A recent study found that children who experienced persistent combined wetting had a much higher chance of bedwetting in adolescence than those with bedwetting alone [28]. Identification of factors that distinguish between different types of UI could increase understanding of the aetiology of childhood incontinence and help to identify children at risk of persistent UI. In this study, we use data from a large cohort to prospectively examine whether biopsychosocial factors are differentially associated with bedwetting, daytime wetting and combined wetting in primary school-aged children.

## Methods

### Participants

The sample comprised participants from the Avon Longitudinal Study of Parents and Children (ALSPAC). Detailed information is available at (http://www.bristol.ac.uk/alspac), including a fully searchable data-dictionary (http://www.bris.ac.uk/alspac/researchers/data-access/data-dictionary). Pregnant women resident in the former Avon Health Authority in SW England having an estimated date of delivery between 1/4/91 and 31/12/92 were invited to take part, resulting in a cohort of 14,541 pregnancies [29]. Of the 13,978 singletons/twins alive at 1 year, a small number of participants withdrew consent (*n* = 24) leaving a starting sample of 13,954. Ethical approval was obtained from the ALSPAC Law and Ethics committee and local research ethics committees.

### Parallel latent classes of bedwetting and daytime wetting

We used parallel latent classes previously extracted from ALSPAC data on bedwetting and daytime wetting at 4^1^/_2_, 5^1^/_2_, 6^1^/_2_, 7^1^/_2_ and 9^1^/_2_ (hereafter referred to as 4–9 years) [28]. The classes were described as ‘normative’ (low probability of bedwetting or daytime wetting at any time point: 63.0% of the sample), ‘daytime wetting alone’ (daytime wetting with very low probability of bedwetting: 5.8%), ‘bedwetting alone’ (bedwetting with very low probability of daytime wetting: 15.6%), ‘delayed’ (decreasing probability of bedwetting or daytime wetting after 6 years: 8.6%) and ‘persistent wetting’ (relatively high probability of bedwetting and daytime wetting at all time points: 7.0%). The latter two classes comprised combined day and night wetting. Boys were more likely to be members of the bedwetting alone (68.4%), persistent wetting (63%) and delayed (52.9%) classes than girls, whilst girls were more likely to be members of the daytime wetting alone class (66.5%) [28].

### Biopsychosocial factors

We examined biological and developmental factors (developmental level at 18 months [30], birth weight, gestation, parental history of UI [31]) and psychosocial factors (child temperament at 2 years [32, 33], behaviour/emotional problems [34] and temper tantrums at 3½ years, exposure to stressful events between 2½ and 3 years 11 months [22], maternal antenatal depression at 18 and 32 weeks gestation and postnatal depression when the child was 21 months [35, 36]). We also examined age at initiation of toilet training [37] and the presence of constipation at age 4 (see table S1 for details of all assessments).

### Confounders

We adjusted for child’s sex and measures of socioeconomic position and adversity derived from responses to questionnaires completed by mothers during the antenatal period (Table 1). We also adjusted for developmental level and maternal depression (full details of the adjustments for each model are provided in the footnote for Table 2).

**Table 1:** Distribution of socio-demographic factors across the UI latent classes (*n* = 8751)

	Normative (%)	Bedwetting alone (%)	Daytime wetting alone	Delayed	Persistent wetting	p
Maternal educational qualifications^a^						
None	25.0	20.4	22.7	21.6	20.8	0.002
Parental social class^b^						
Manual	15.5	14.7	17.9	13.9	17.4	0.361
Family size						
≥ 3 children	0.9	1.1	1.0	1.6	1.3	0.345
Early parenthood (< 19 years)						
Yes	4.8	4.8	4.4	3.9	5.8	0.639
Housing adequacy^c^						
No	4.9	3.7	4.4	5.3	6.4	0.165
Major financial difficulties						
Yes	8.1	7.7	10.5	8.7	10.7	0.145

We obtained these distributions by assigning each participant to their most likely class (modal assignment), generating individual probabilities of class membership and deriving conditional distributions of the variables within classes

^a^Maternal education defined as none versus high school qualifications or greater

^b^Social class based on the higher of the mother or partner’s occupational social class using the 1991 British Office of Population and Census Statistics (OPCS) classification and dichotomized into non-manual (professional, managerial or skilled professions) and manual (partly or unskilled occupations)

^c^Housing adequacy: ‘no’ comprises crowding, periods of homelessness, poor living conditions and/or major defects/infestation

**Table 2:** Odds ratios and 95% confidence intervals for the association between the biopsychosocial factors and latent class membership (*n* = 8751)

	*N*	Bedwetting alone	Daytime wetting alone	Delayed	Persistent wetting	*p* value
Biological and developmental factors						
Developmental level at 18 months^a^	7301	1.04 [0.94–1.15]	1.43 [1.20–1.70]	1.52 [1.33–1.72]	1.35 [1.18–1.55]	< 0.001
Gestational age < 37 weeks (ref = ≥ 37 weeks)^b^	7771	0.87 [0.55–1.38]	0.84 [0.32–2.21]	1.48 [0.90–2.43]	1.17 [0.70–1.93]	0.511
Birth weight < 2.5 kg (ref = ≥ 2.5 kg)^b^	7622	0.97 [0.59–1.59]	2.35 [1.27–4.35]	1.12 [0.57–2.17]	1.14 [0.64–2.04]	0.081
Maternal history of bedwetting after age 5 (ref = none)^c^	8516	2.75 [2.08–3.64]	1.31 [0.82–2.38]	1.66 [1.07–2.56]	3.77 [2.81–5.06]	0.001
Maternal history of daytime wetting after age 5 (ref = none)^c^	8516	0.31 [0.03–3.66]	2.41 [0.59–9.87]	2.35 [0.92–6.03]	3.60 [1.75–7.40]	0.004
Paternal history of bedwetting after age 5 (ref = none)^c^	6296	1.67 [1.20–2.32]	1.49 [0.84–2.66]	1.57 [1.04–2.37]	1.53 [1.00–2.33]	0.002
Paternal history of daytime wetting after age 5 (ref = none)^c^	6296	0.95 [0.06–15.13]	2.91 [0.32–26.47]	8.82 [3.01–25.8]	2.34 [0.33–16.81]	0.002
Psychosocial factors						
Difficult temperament at 2 years^d^						
Activity	6613	1.13 [1.03–1.25]	1.07 [0.91–1.26]	1.27 [1.12–1.44]	1.03 [0.91–1.17]	< 0.001
Adaptability	6568	1.17 [1.06–1.29]	1.21 [1.02–1.45]	1.27 [1.12–1.44]	1.25 [1.10–1.41]	< 0.001
Intensity	6390	1.07 [0.97–1.18]	1.04 [0.87–1.26]	1.18 [1.01–1.37]	1.24 [1.09–1.41]	< 0.001
Mood	6590	1.12 [1.01–1.23]	1.34 [1.13–1.58]	1.33 [1.18–1.51]	1.26 [1.11–1.44]	< 0.001
Persistence	6508	1.09 [0.99–1.21]	1.04 [0.87–1.24]	1.15 [1.00–1.33]	1.21 [1.06–1.38]	0.003
Behavior/emotional problems at 3½ years^e^						
Emotional difficulties	6696	1.04 [0.95–1.14]	1.11 [0.94–1.32]	1.21 [1.06–1.38]	1.20 [1.06–1.36]	< 0.001
Conduct difficulties	6696	1.17 [1.06–1.29]	1.29 [1.08–1.54]	1.61 [1.42–1.82]	1.28 [1.14–1.44]	< 0.001
Hyperactivity	6696	1.05 [0.95–1.15]	1.05 [0.88–1.25]	1.37 [1.22–1.55]	1.24 [1.11–1.39]	< 0.001
Low level of prosocial behaviour	6696	1.02 [0.92–1.13]	1.05 [0.87–1.28]	1.39 [1.21–1.59]	1.17 [1.03–1.32]	< 0.001
Total behaviour difficulties	6696	1.20 [1.08–1.32]	1.33 [1.12–1.59]	1.80 [1.59–2.03]	1.56 [1.39–1.74]	< 0.001
Temper tantrums at least once a day at 3½ years (ref = once a week or rarer)^e^	6644	1.15 [0.88–1.52]	1.26 [0.80–2.00]	1.37 [0.97–1.96]	1.55 [1.13–2.13]	0.010
Stressful events between 2½ and 3 years 11 months	6626	1.05 [0.94–1.17]	1.25 [1.05–1.49]	1.20 [1.04–1.39]	1.26 [1.11–1.43]	< 0.001
Maternal depression						
Antenatal (18 weeks)^f^	7345	1.18 [0.88–1.60]	0.96 [0.54–1.69]	1.03 [0.66–1.63]	1.47 [1.05–2.07]	0.166
Antenatal (32 weeks)^f^	7711	1.29 [0.98–1.69]	1.27 [0.79–2.02]	1.31 [0.89–1.92]	1.29 [0.93–1.78]	0.085
Postnatal (21 months)^g^	6880	1.30 [0.93–1.83]	2.38 [1.46–3.88]	1.19 [0.69–2.03]	2.09 [1.48–2.95]	< 0.001
Toilet training and constipation						
Age at initiation of toilet training (ref = 15–24 months)^h^						
Before 6 months	6615	0.78 [0.36–1.69]	0.71 [0.15–3.26]	0.31 [0.02–4.06]	1.08 [0.45–2.60]	0.001
6–15 months		1.12 [0.84–1.49]	1.31 [0.78–2.20]	0.89 [0.54–1.48]	1.10 [0.74–1.64]	
After 24 months		1.01 [0.82–1.25]	1.73 [1.18–2.55]	1.58 [1.18–2.10]	1.59 [1.19–2.21]	
Constipation at 4 years 9 months (ref = none)^i^	6624	0.82 [0.60–1.11]	1.29 [0.74–2.27]	1.49 [1.06–2.09]	1.14 [0.81–1.61]	0.035

The exact sample size for each analysis differed depending on the availability of non-missing data on biopsychosocial factors and confounders. Odds ratios for these analyses were derived in relation to the normative class. For the continuous variables (developmental level, difficult temperament, psychological problems, stressful events), results show the increase in odds of membership to each latent class per one standard deviation (SD) increase in the score

^a^Adjusted for child’s sex (0 = female vs. 1 = male), socioeconomic variables (parental social class [0 = non-manual: professional, managerial or skilled professions vs. 1 = manual: partly or unskilled occupations], early parenthood [0 = child born when mother was >=19 years vs. 1 = child born when mother was < 19 years], maternal education [0 = high school qualifications or greater vs. 1 = vocational or none], housing adequacy [0 = yes vs. 1 = no: comprising crowding, periods of homelessness, living conditions, major defects/infestation], major financial difficulties [0 = no vs. 1 = yes], family size [0 = less than 3 children vs. 1 = 3 or more children], presence of a social network [0 = presence of network vs. 1 = no network: comprising lack of emotional, practical and/or financial support], maternal antenatal depression (32 weeks) and postnatal depression (21 months)

^b^Adjusted for child’s sex, socioeconomic variables, and maternal antenatal depression (32 weeks)

^c^Adjusted for child’s sex

^d^Adjusted for child’s sex, socioeconomic variables, developmental level of child at 18 months, and maternal postnatal depression (21 months)

^e^Adjusted for child’s sex, socioeconomic variables, developmental level of child at 18 months, and maternal postnatal depression (33 months)

^f^Adjusted for child’s sex and socioeconomic variables

^g^Adjusted for child’s sex, socioeconomic variables, and maternal antenatal depression (18 weeks)

^h^Adjusted for child’s sex, socioeconomic variables, developmental level of child at 18 months, and maternal postnatal depression (21 months)

^i^Adjusted for child’s sex, socioeconomic variables, developmental level of child at 18 months, and maternal postnatal depression (33 months)

### Statistical modelling

We estimated the association between the biopsychosocial factors and the latent classes using a series of univariable multinomial logistic regression models and employing the normative latent class as the baseline category for the outcome. Parameter estimates were obtained using the “Modal ML” 3-step method [38] implemented in Mplus. This has been shown to produce less-biased estimates than traditional three-step methods such as probability weighting, whilst avoiding the problem of covariates impacting on the measurement model itself [39]. Bias-adjusted estimates were obtained using the Mplus “auxiliary (r3step)” command.

## Results

We focused on the sample of children (*n* = 8751, 4507 boys, 4244 girls) with bedwetting and daytime wetting data available for at least three of the five time points. Table 1 shows the distribution of socio-demographic variables across the latent classes. Apart from maternal education, there was little evidence of an association between the socio-demographic factors and childhood UI. Table 2 presents the results of the adjusted analysis examining the associations between the biopsychosocial factors and membership of the latent classes (see table S2 for unadjusted results).

### Biological and developmental factors

Developmental delay at 18 months was strongly associated with increased odds of daytime wetting alone, delayed and persistent wetting, but not bedwetting alone. Low birth weight was associated with daytime wetting alone, but gestational age was not associated with childhood UI. Maternal history of bedwetting after age 5 was strongly associated with bedwetting alone, delayed bladder control and persistent wetting (associations were stronger than those found for paternal history of bedwetting). Maternal history of daytime wetting after age 5 was associated with persistent wetting, whilst paternal history of daytime wetting was associated with delayed bladder control. Child’s male sex was an important confounder for bedwetting alone, delayed and persistent wetting classes, whilst female sex was an important confounder for daytime wetting alone.

### Psychosocial factors

There was an association between difficult temperament (especially adaptability and mood) and childhood UI. There was evidence that behaviour/emotional problems were associated with UI; the strongest associations were found for the delayed class. Emotional difficulties, hyperactivity and prosocial behaviour were only associated with the delayed and persistent wetting classes, whilst there was no evidence of an association with either bedwetting or daytime wetting alone. Conduct difficulties and total behaviour problems were associated with all the UI classes. There was strong evidence that temper tantrums in early childhood were associated with persistent wetting. In these analyses, the most important confounders were male sex (bedwetting alone, persistent wetting), female sex (daytime wetting alone), developmental delay and maternal postnatal depression.

It is notable that stressful events were associated with all the UI classes except bedwetting alone. There was some evidence of confounding when persistent wetting was the outcome (male sex, developmental delay and maternal postnatal depression were the most important confounders).

Antenatal maternal depression at 18 weeks (but not at 32 weeks) was associated with persistent wetting (male sex, maternal education and housing inadequacy were confounders). Postnatal depression was strongly associated with daytime wetting alone and persistent wetting (female sex was a confounder in the analysis with daytime wetting).

### Toilet training and constipation

Initiation of toilet training after 24 months was strongly associated with daytime wetting alone, delayed bladder control and persistent wetting at school age, but not bedwetting alone. Confounders were male sex (female sex in analysis with daytime wetting alone), developmental delay and maternal postnatal depression.

Constipation in early childhood was associated with delayed attainment of bladder control after adjusting for confounders (developmental delay was the most influential confounder). Constipation was associated with persistent wetting in the unadjusted, but not the adjusted model (confounders were male sex, housing inadequacy, developmental delay and maternal postnatal depression).

## Discussion

### Main findings

This is the first study to prospectively examine a wide range of biopsychosocial factors in relation to different trajectories of childhood UI. Parental history of bedwetting after age 5 was strongly associated with bedwetting alone, delayed and persistent trajectories of wetting in offspring (the association was strongest for maternal bedwetting). Maternal history of daytime wetting was associated with persistent wetting, whilst paternal daytime wetting was associated with delayed bladder control in offspring. In general, difficult temperament and behaviour/emotional problems were most strongly associated with the combined trajectories of childhood UI (delayed and persistent wetting). Maternal (antenatal and postnatal) depression was associated with persistent wetting and postnatal depression was associated with daytime wetting. Developmental delay, stressful events, and later initiation of toilet training were not associated with bedwetting alone, but were strongly associated with the other trajectories of childhood UI.

### Strengths and limitations

Major strengths of this study include the prospective design and use of parallel latent classes of UI derived from repeated measures of bedwetting and daytime wetting in a large cohort. Another strength is the availability of a wide range of biopsychosocial factors and extensive data on confounders. A possible limitation is the use of maternal reports of difficult temperament and behaviour/emotional problems since parents of children with UI may be more negatively predisposed to their children and, hence, more likely to report these problems. However, bedwetting is not normally considered problematic in early childhood (at the time of assessing these problems), reducing the likelihood of biased maternal assessments. We are also limited by having only five time points when parents were asked about their child’s UI.

### Comparison with earlier findings

#### Biological and developmental factors

We found evidence that developmental delay at 18 months was associated with daytime wetting alone, delayed bladder control and persistent wetting, but not bedwetting alone. Earlier studies reported that maturational deficits are linked to bedwetting [2–7], but they did not differentiate between bedwetting with or without daytime wetting. One small study, comparing rates of delayed development in children with bedwetting, daytime wetting and mixed day and night wetting to controls, found that children with bedwetting had higher rates of delayed development than controls [3]. We found no evidence that gestational age is associated with childhood UI, but low birth weight was linked to daytime wetting. Earlier studies found that low birth weight children were more likely to have UI [4, 5].

Maternal history of bedwetting was strongly associated with bedwetting alone, delayed attainment of bladder control and persistent wetting in offspring. Weaker associations were found for paternal history of bedwetting. We also found associations between parental history of daytime wetting and offspring UI. The association between parental and offspring UI was previously examined in the ALSPAC cohort, but childhood UI was examined at a single time point (age 7½) and the study did not exclude children with daytime wetting from the bedwetting group and vice versa, nor did they examine the group with combined wetting separately [31]. Previous studies have shown that bedwetting is highly familial [2–5] and one study found a familial influence for both bedwetting and daytime wetting.^3^ In comparison to our finding of a stronger association for maternal bedwetting and offspring UI, a previous study found that paternal bedwetting history had a stronger association with offspring bedwetting, but the confidence intervals overlapped [4].

#### Psychosocial factors

Child temperament and behaviour/emotional problems generally had the strongest associations with the combined trajectories of UI (delayed bladder control and persistent wetting) and the weakest associations with bedwetting alone. Previous studies have found higher levels of psychological problems in children with combined wetting, but these were cross-sectional [26, 27]. It is notable that the hyperactivity scale was only associated with the combined trajectories of UI. Previous studies have reported higher rates of ADHD in children with bedwetting and daytime wetting [40] but the majority are cross-sectional and relied on parental reports, few distinguished between different trajectories of UI and some did not have a comparison group of children without UI.

It is commonly believed that bedwetting is associated with stress, but we found that stressful events are associated with all the UI classes except bedwetting alone. Two earlier prospective studies examined this link but they did not distinguish between bedwetting, daytime wetting alone and combined wetting [11, 22]. We did not distinguish between life events that are acute (e.g. bereavement) versus chronic (e.g. financial problems) or between normative (e.g. birth of a sibling) and non-normative (unpredictable) stressors (e.g. accidents). It is difficult to isolate the effect of single types of stressful events because they are often interrelated.

There was strong evidence that exposure to postnatal depression is associated with daytime wetting alone and persistent wetting. There was also evidence for an association between antenatal depression (at 18 weeks) and persistent wetting. Earlier studies have reported a link between maternal psychopathology and bedwetting, but they did not distinguish between bedwetting with or without daytime wetting [10, 33]. One study examined only parental lifetime psychopathology and did not separately examine maternal psychopathology or the timing of the disorder (antenatal or postnatal) [10]. The other study only examined exposure to maternal psychopathology in the postnatal period [33].

#### Toilet training and constipation

We found strong evidence that later initiation of toilet training is associated with daytime and combined wetting, but not bedwetting alone. This is consistent with an earlier study reporting that the development of nocturnal bladder control is a maturational process that is not affected by parental potty training practices [13]. Previous studies have found that delayed or inadequate toilet training is associated with bladder dysfunction (e.g. urgency, urge incontinence, emptying difficulties, bladder instability and/or dyscoordinated micturition) [14–16]. Another study found that early toilet training (before 18 months) was associated with a lower rate of daytime wetting and bedwetting [41].

We found that constipation in early childhood is only associated with delayed bladder control at school age. Constipation has frequently been found to be associated with childhood UI, but the majority of the studies are cross-sectional [17–19].

### Possible mechanisms explaining the associations between the biopsychosocial factors and childhood UI

The current findings provide evidence for differential associations between biopsychosocial factors and bedwetting alone, daytime wetting alone and combined wetting. Developmental delay was related to daytime wetting alone and combined (day and night) wetting, but not with bedwetting alone. According to the ICCS classification, monosymptomatic nocturnal enuresis (bedwetting alone) is attributed to nocturnal polyuria and a deficit in the basic inhibitory function of the brainstem [24] while non-monosymptomatic nocturnal enuresis (bedwetting accompanied by daytime urinary symptoms) is often attributed to overactive bladder [25]. Bedwetting has been described as a genetically determined maturational disorder of the CNS [7] and, consistent with earlier studies, we find evidence for a strong association between parent and offspring bedwetting. Compared with the combined trajectories of childhood UI, bedwetting alone generally had weaker associations with early behaviour/emotional problems and was not associated with exposure to early stressful life events or maternal depression. Additionally, age at initiation of toilet training was not associated with bedwetting alone, but was associated with daytime and combined trajectories of UI. This suggests that external stressors or toilet training practices do not affect processes relating to nocturnal bladder control and that bedwetting alone is more strongly related to inherited factors.

We found strong associations between maternal history of bedwetting and offspring bedwetting alone and combined (day and night) wetting in offspring. Maternal-offspring associations were stronger than those found for paternal history of wetting and the most likely reason for this is the Carter effect [42]. Bedwetting is mainly transmitted by polygenic inheritance and males are more affected than females [43]. The less commonly affected sex (females) will carry a higher load of genes; therefore, the strength of the mother–offspring association will be greater than the father–offspring association. The associations we found between parental history of daytime wetting and offspring UI were less consistent. Few studies have examined heritability of daytime wetting because daytime wetting is even more heterogeneous than bedwetting [43] and environmental factors are thought to play a greater role in some subtypes of daytime wetting [44].

Exposure to maternal depression in the antenatal (18 weeks) and postnatal periods was associated with persistent wetting and postnatal depression was related to daytime wetting alone. Antenatal depression is linked to higher cortisol levels in offspring [45] and HPA axis alterations are associated with adverse developmental outcomes in offspring [46]. Exposure to postnatal depression at 21 months coincides with a peak period of toilet training initiation in this cohort [37]. At around this age, there is thought to be a sensitive period for the attainment of bladder control and stress can interfere with this process [12]. The association with postnatal depression could be due to depressed mothers being less able to cope with the demands of toilet training and less responsive to their child’s toileting needs which, in turn, may increase the child’s stress levels. Further studies are needed with detailed data on toilet training practices to allow examination of the relationship between maternal depression and offspring behaviour/emotional problems in influencing toilet training outcomes.

The associations we report here could also be due to a shared genetic susceptibility for negative affect since there is strong evidence for a link between affective symptoms and lower urinary tract symptoms, particularly overactive bladder [47]. Further research is needed to determine whether there is a causal association between affective symptoms and urinary symptoms.

Constipation was only associated with delayed attainment of bladder control. Previous studies have found that constipation is more common in children with developmental delay [48], which could explain our finding.

## Conclusion

We find evidence that different trajectories of childhood UI are differentially associated with biopsychosocial factors. This knowledge could inform medical history taking and potentially aid the identification of children at risk of persistent continence problems. Our finding of prospective associations between psychological problems and continence problems has important clinical implications. It is important to know about associations between psychological factors and UI, because these factors can affect treatment adherence and persistence of continence problems could be due to untreated psychological factors [49]. There is a need for an increased understanding of the role of psychological factors in relation to continence problems and further research is needed to determine whether these associations are likely to be causal. Prospective associations are not definitive evidence of causal effects because residual confounding and reverse causation are key problems in observational epidemiology. If causal effects exist between psychological factors and UI, treatments aimed at reducing psychological problems in children and mothers could lead to a reduction in childhood continence problems.

## Electronic supplementary material

Below is the link to the electronic supplementary material.

Supplementary material 1 (DOCX 117 kb)
