# Supplementary material for: A prospective cohort study of biopsychosocial factors associated with childhood urinary incontinence
Source: Eur Child Adolesc Psychiatry. 2018 Jul 6;28(1):123–30. doi: 10.1007/s00787-018-1193-1 (PMC6349792; doi:10.1007/s00787-018-1193-1)
Supplement: Supplementary file 1 — Supplementary material 1 (DOCX 117 kb) [file 787_2018_1193_MOESM1_ESM.docx]

**Supplementary materials**

Table S1. Details of biopsychosocial factors assessed in early childhood

|  | **Risk factor and age of child at assessment** | **Assessment** | **Details of how it was analysed** |
| --- | --- | --- | --- |
| Biological and developmental factors | Developmental level at 18 months | Parent-reported questionnaire developed by ALSPAC including items from the Denver Developmental Screening Test^29^ | We used a total development score adjusted for age in weeks, standardized (using a linear regression model and extracting the residuals) and reversed where appropriate so that high values on all scores reflected a lower level of development. |
|  | Birth weight | Recorded from obstetric notes by ALSPAC staff at maternity hospitals | Low birth weight was defined as less than 2.5kg consistent with earlier studies^1,5^ |
|  | Gestation | Derived from date of delivery, date of last menstrual period and ultrasound data where available. | Preterm birth was indicated by gestation less than 37 weeks. |
|  | Parental history of bedwetting and daytime wetting after age 5 | Obtained from self-report questionnaires^30^ | We derived binary variables indicating whether mothers or fathers had any versus no history of wetting (excluding isolated occurrences of maternal bedwetting around the perinatal period). |
| Psychosocial factors | Child temperament at 2 years | Mothers completed the Toddler Temperament Scale (TTS).^31^ The TTS comprises statements describing specific behaviours and mothers were asked to rate how often their child behaves in that way on a scale ranging from 1 (almost never) to 6 (almost always). | The scale comprises nine temperament traits, but we restricted our analysis to the five traits (activity, adaptability, intensity, mood, persistence) that were associated with bedwetting in an earlier study.^32^ We standardized the score for each temperament trait. |
|  | Behaviour/emotional problems at 3½ years | Mothers completed the Revised Rutter Parent Scale for Preschool Children^33^ comprising emotional and conduct difficulties, hyperactivity and prosocial behaviour. | Responses were aggregated to create scores in the four domains. High levels of psychological problems are indicated by high scores on emotional difficulties, conduct difficulties and hyperactivity and low scores on the prosocial behaviour scale. We standardized the score for each behavior/emotional problem. |
|  | Temper tantrums at 3½ years | Mothers were asked the question “How often does he/she have temper tantrums?” with response options: more than once a day; most days; at least once a week; less than once a week; never. | We derived a binary variable indicating whether temper tantrums occurred at least once a day (coded 1) versus once a week or less (coded 0). |
|  | Stressful events between 2½ years & 3 years 11 months | Mothers completed a 42-item questionnaire when the study child was 3 years 11 months, asking “Have any of these [events] occurred since the study child was 2 ½ years old?”^21^ | We generated a total life events score by adding all events (presence of life event =1, absence = 0) and then standardized the score. |
|  | Maternal antenatal (18 weeks and 32 weeks gestation) and postnatal (21 months) depression | Mothers completed the Edinburgh Postnatal Depression Scale (EPDS).^34^ | We dichotomized the EPDS at the standard cut-off (score>12) used to indicate probable depressive disorder.^35^ |
| Toilet training and constipation | Age at initiation of toilet training | Mother completed questionnaires when their child was 6, 15, and 24 months including a question asking whether they had initiated toilet training. | Responses to the questions were used to create a 4-level variable indicating whether toilet training had been initiated before 6 months, between 6 and 15 months, between 15 and 24 months, or after 24 months.^36^ |
|  | Constipation at 4 years 9 months | Mothers were asked about their child’s constipation: “Has he/she had any constipation in the past 12 months?” Options were: ‘Yes, and saw a doctor’, ‘Yes, but did not see the doctor’, and ‘No, did not have’. | We derived a binary variable indicating the presence or absence of constipation. |

Table S2. Odds ratios and 95% confidence intervals for the association between the biopsychosocial factors and latent class membership (**unadjusted results**).

|  | **Bedwetting alone** | **Daytime wetting alone** | **Delayed** | **Persistent wetting** | p-value |
| --- | --- | --- | --- | --- | --- |
| ***Biological and developmental factors*** |  |  |  |  |  |
| Developmental level at 18 months | 1.19 [1.09-1.29] | 1.37 [1.16-1.62] | 1.44 [1.28-1.63] | 1.53 [1.36-1.73] | <0.001 |
| Gestational age <37weeks (ref= >=37weeks) | 0.97 [0.66-1.43] | 1.02 [0.50-2.04] | 1.47 [0.95-2.27] | 1.19 [0.76-1.86] | 0.415 |
| Birth weight < 2.5kg (ref= >=2.5kg) | 1.04 [0.69-1.58] | 2.66 [1.59-4.44] | 1.21 [0.70-2.10] | 1.12 [0.68-1.87] | 0.004 |
| Maternal history of bedwetting after age 5 (ref= none) | 2.64 [2.01-3.47] | 1.30 [0.70-2.41] | 1.68 [1.09-2.59] | 3.68 [2.75-4.91] | <0.001 |
| Maternal history of daytime wetting after age 5 (ref=none) | 0.30 [0.02-4.08] | 2.61 [0.78-8.75] | 2.24 [0.86-5.84] | 3.57 [1.74-7.33] | 0.004 |
| Paternal history of bedwetting after age 5 (ref= none) | 1.62 [1.18-2.22] | 1.50 [0.84-2.70] | 1.58 [1.04-2.39] | 1.49 [0.98-2.26] | 0.002 |
| Paternal history of daytime wetting after age 5 (ref= none) | 1.01 [0.08-12.1] | 2.93 [0.24-35.5] | 8.60 [2.92-25.3] | 2.61 [0.44-15.4] | 0.003 |
|  |  |  |  |  |  |
| ***Psychosocial factors*** |  |  |  |  |  |
| Difficult temperament at 2 years |  |  |  |  |  |
| Activity | 1.18 [1.08-1.29] | 1.08 [0.92-1.25] | 1.10 [0.99-1.23] | 1.23 [1.09-1.38] | <0.001 |
| Adaptability | 1.18 [1.09-1.29] | 1.23 [1.06-1.43] | 1.34 [1.21-1.50] | 1.35 [1.21-1.50] | <0.001 |
| Intensity | 1.06 [0.97-1.16] | 1.06 [0.90-1.25] | 1.27 [1.13-1.42] | 1.21 [1.06-1.37] | <0.001 |
| Mood | 1.11 [1.01-1.21] | 1.37 [1.19-1.58] | 1.33 [1.19-1.49] | 1.32 [1.18-1.47] | <0.001 |
| Persistence | 1.12 [1.03-1.22] | 1.15 [0.99-1.34] | 1.30 [1.17-1.46] | 1.33 [1.17-1.50] | 0.003 |
| Psychological problems at 3½ years |  |  |  |  |  |
| Emotional difficulties | 1.00 [0.92-1.09] | 1.20 [1.05-1.38] | 1.27 [1.15-1.40] | 1.24 [1.11-1.38] | <0.001 |
| Conduct difficulties | 1.22 [1.12-1.33] | 1.29 [1.10-1.51] | 1.41 [1.29-1.56] | 1.63 [1.46-1.81] | <0.001 |
| Hyperactivity | 1.07 [0.98-1.16] | 1.10 [0.95-1.27] | 1.35 [1.22-1.50] | 1.48 [1.32-1.65] | <0.001 |
| Low level of prosocial behaviour | 1.12 [1.03-1.22] | 1.17 [1.01-1.34] | 1.32 [1.19-1.47] | 1.51 [1.35-1.68] | <0.001 |
| Total behaviour difficulties | 1.22 [1.12-1.33] | 1.46 [1.26-1.70] | 1.71 [1.55-1.88] | 1.83 [1.65-2.03] | <0.001 |
| Temper tantrums at least once a day at 3½ years (ref= once a week or rarer) | 1.16 [0.91-1.48] | 1.40 [0.94-2.08] | 1.75 [1.35-2.25] | 1.43 [1.06-1.93] | <0.001 |
| Stressful events at 3½ years | 1.03 [0.94-1.12] | 1.22 [1.05-1.42] | 1.25 [1.13-1.39] | 1.14 [1.01-1.27] | <0.001 |
| Maternal depression |  |  |  |  |  |
| Antenatal (18 weeks) | 1.11 [0.85-1.46] | 1.09 [0.66-1.79] | 1.07 [0.73-1.58] | 1.65 [1.23-2.20] | 0.015 |
| Antenatal (32 weeks) | 1.15 [0.90-1.47] | 1.21 [0.78-1.87] | 1.20 [0.86-1.69] | 1.38 [1.03-1.85] | 0.100 |
| Postnatal (21 months) | 1.23 [0.90-1.67] | 2.23 [1.44-3.43] | 1.40 [0.93-2.10] | 2.11 [1.54-2.88] | <0.001 |
|  |  |  |  |  |  |
| ***Toilet training and constipation*** |  |  |  |  |  |
| Age at initiation of toilet training (ref= 15–24 months): |  |  |  |  |  |
| Before 6 months | 0.63 [0.33-1.19] | 0.64 [0.19-2.21] | 0.67 [0.27-1.65] | 0.26 [0.04-1.75] | <0.001 |
| 6 – 15 months | 0.95 [0.74-1.23] | 1.07 [0.67-1.73] | 1.19 [0.85-1.67] | 0.76 [0.48-1.19] |  |
| After 24 months | 1.17 [0.97-1.42] | 1.54 [1.09-2.17] | 1.95 [1.54-2.47] | 1.78 [1.38-2.29] |  |
| Constipation at 4 years 9 months (ref= none) | 0.82 [0.63-1.08] | 1.43 [0.96-2.14] | 1.12 [0.83-1.51] | 1.47 [1.10,1.96] | 0.006 |
